# Exosomal lncRNA HCG18 contributes to cholangiocarcinoma growth and metastasis through mediating miR-424-5p/SOX9 axis through PI3K/AKT pathway

**DOI:** 10.1038/s41417-022-00500-2

**Published:** 2023-02-28

**Authors:** Qingfeng Ni, Hai Zhang, Xiaoli Shi, Xiangcheng Li

**Affiliations:** The First Affiliated Hospital of Nanjing Medical University, The National Institute of Living Donor Liver Transplantation, Nanjing, Jiangsu 210029 P. R. China

**Keywords:** Cancer, Cancer

## Abstract

Cholangiocarcinoma is a highly aggressive malignant tumor disease with the increasing incidence and mortality. It’s urgent to identify specific biomarkers for cholangiocarcinoma treatment and diagnosis. Recent studies have noted the importance of lncRNAs in cancer and the following downstream mechanism with miRNAs network has been a hotspot. This work aimed to discover the role of lncRNA HCG18 and its possible downstream mechanism in cholangiocarcinoma tumor progression. Initially, through bioinformatics tools, we observed abnormal expression of lncRNA HCG18 in cholangiocarcinoma. In vitro experiments like (CCK-8, EdU, colony formation, flow cytometry, transwell, wound healing assays) and animal study confirmed that lncRNA HCG18 served as a cancer-promoting gene, promoted cancer proliferation, migration and invasion abilities. Besides, we found cancer cell-secreted exosomes transitted HCG18 to surrounding tumor cells and accelerated tumor growth and metastasis. After that, we confirmed HCG18 directly interacted with miR-424-5p through FISH, RIP and dual luciferase reporter assays with negative modulation. The inhibition of miR-424-5p reversed the HCG18 knockdown induced suppression on cholangiocarcinoma cancer cells. More specific, miR-424-5p targeted to SOX9 contributed to cholangiocarcinoma growth and metastasis through mediating PI3K/AKT pathway. In conclusion, these findings provide solid evidence of lncRNAs/miRNAs regulation in cholangiocarcinoma progression.

## Introduction

Cholangiocarcinoma is a highly aggressive malignant tumor disease originating from bile duct epithelial cells, with the increasing incidence and mortality [1]. Cholangiocarcinoma is characterized by high malignancy, insidious onset and difficult diagnosis and treatment [2, 3]. Although radical surgical resection is currently the first choice for clinical treatment of cholangiocarcinoma, lack of postoperative prognosis of patients is urgency [4]. Molecular targeted therapy has been a hot spot in recent tumor therapy area due to its strong specificity and less side effects. However, currently, specific biomarker for cholangiocarcinoma is rarely explored, so more in-depth studies are needed to explore the molecular mechanism of the occurrence and progression of cholangiocarcinoma.

Long non-coding RNAs (lncRNAs), existing in eukaryotes, are essential group members of non-coding RNAs. The length of lncRNAs is more than 200 nt and no encoding protein ability is their specific feature. Compared with mRNAs, lncRNAs are lack of cap or polyA [5]. They exert their functions by the secondary structures and interactions with corresponding target miRNAs. Generally speaking, as necessary material basis, lncRNAs coordinate and complete the complex regulatory process of life. Abundant researches uncovered that the occurrence and development of cancer can be mediated by a variety of mechanisms of lncRNAs, mainly through epigenetic regulation, activation of carcinogenic pathways and interference of other RNA subtypes, which meant lncRNAs can be seen as prospective targets of cancer [6–8]. LncRNA HLA complex group 18 (lncRNA HCG18) has been recognized as a cancer-promoting gene in various cancers by existing researches. For instance, lncRNA HCG18 overexpressed in hepatocellular carcinoma and promotes tumor development by regulating miR-214-3p/CENPM [9]. Besides, increasing HCG18 level was also associated with colorectal cancer and nasopharyngeal carcinoma through competing endogenous RNAs (ceRNAs) networks [10, 11]. The role of HCG18 in cholangiocarcinoma still remains unknown.

Exosomes are small microvesicles contain functional mRNAs, miRNAs or lncRNAs, which can fuse with cell membrane and release the small vesicles [12, 13]. Although exosomes were initially considered to be “garbage” of cells and therefore discharged, recent studies have shown that exosomes have functional activity and can carry out information transmission between cells, tightly participate in antigen presentation process, tumor development, and nerve cell signal transduction [14, 15]. It is reported that exosomal lncRNA 00152 is enriched in gastric cancer cells plasma [16]. Elevating HOTAIR level was detected in urine exosomes of patients with bladder cancer and is associated with local tumor transition [17]. The evidence supported that exosomal lncRNAs are confirmed as novel biomarkers in cancer.

As major functional parts in ceRNAs network, down-regulating target mRNAs expression in cytoplasm by binding 3’-UTR in seed region is one of the miRNAs typical biological functions. Recent studies have found that miRNA expression is closely associated with tumorigenesis, and about 50% of the annotated miRNAs are localized at tumor-associated fragile sites, served as tumor suppressor genes or oncogenes [18, 19]. MiR-424-5p, has been identified as a tumor suppressor, involved in inhibiting cancer growth and metastasis in intrahepatic cholangiocarcinoma and breast cancer [20, 21]. However, much of the research up to now has also descripted that miR-424-5p level is increasing in colon cancer and pancreatic cancer [22, 23]. Therefore, the role of miR-424-5p in cancer development still remains inconsistent. Sox9, SRY-box transcription factor 9, was found expressed abnormally in various cancers in many studies but its relevant mechanism was hardly digged out. Sox9 could promote basal cell carcinoma renewal and lead to invasion through Wnt/β-catenin-dependent pathway [24]. Besides, Sox9 was found to participate in the regulation of tumor micro environment (TME) and epithelial-mesenchymal transition (EMT) pathway [25]. Here, we aimed to discover the biological activity of lncRNA HCG18 in cholangiocarcinoma, and the possible interaction between miR-424-5p and Sox9 influencing cancer cell growth and metastasis.

## Materials and methods

### Bioinformatics analysis

The differential expressed lncRNAs and the HCG18 expression in cholangiocarcinoma tumor tissues and in normal tissues were predicted in the TCGA database(https://www.cancer.gov/about-nci/organization/ccg/research/structural-genomics/tcga). The downstream miRNAs with potential binding sites to HCG18 and the target mRNAs of miR-424-5p were predicted by Starbase (https://starbase.sysu.edu.cn/).

### Patient samples collection

Tumor tissues and paracancerous tissue samples were collected from 60 cases of cholangiocarcinoma patients who were diagnosed by histology and none of them accepted chemotherapy or any other treatment at The First Affiliated Hospital of Nanjing Medical University, The National Institute of Living Donor Liver Transplantation. All tissue were stored in liquid nitrogen. Patients were informed of all samples collected and signed informed consent. The research was approved by the ethics committee of The First Affiliated Hospital of Nanjing Medical University.

### Cell culture and transfection

The human cholangiocarcinoma cell line (QBC939, HUCCT1, RBE, HCCC9810) and human intrahepatic bile duct epithelial cell HiBEC were purchased from Shanghai Institute of Cell Biology and incubated in 1640 medium containing 10% FBS and 1% double antibody.

For cell transfection, the cholangiocarcinoma cells (2 × 10^5^ cells/well) were seeded in six-well plates at 37 °C overnight to reach 70% confluence. Small interfering RNA for HCG18 (si-HCG18-1 and -2) and si-HCG18-2, HCG18 overexpression plasmids (HCG18 ov), miR-424-5p mimics, miR-424-5p inhibitors (miR-424-5p inh.), SOX9 overexpression plasmids (SOX9 ov) were provided by GenePharma Co., Ltd. (Shanghai, China). Cells transfected with empty pcDNA3.1 vector (pcDNA3.1-NC), NC mimics (miR-NC) or scrambled siRNA sequences were considered as negative controls (NC). Lipofectamine ^TM^ 3000 (Life Technologies) was used for transfection the knockdown or overexpressed plasmid and their negative control. All steps were following the instruction. Lipofectamine ^TM^ 3000 and plasmids were diluted with serum-free medium, respectively. The diluted Lipofectamine 3000 was added into the corresponding diluted plasmids and incubated at room temperature for 20 min. Serum-free medium was added and cultured at 37 °C and 5% CO_2_ for 6 h. Conventional serum-containing medium was replaced and further cultured for 24 h for subsequent detection.

### Quantitative reverse transcription polymerase reaction (qRT-PCR)

Total RNA was extracted from tissue and cultured cells through the use of the TRizol reagent, and then reverse transcribed into cDNA through the use of the Prime ScriptTM RT reagent Kit. The resulting cDNA was stored at −20 °C for qPCR analysis. The expression of HCG18 and Sox9 was detected by ABI 7900HT RealTime PCR System using SYBR Green assays and GAPDH was the internal control. The expression of miR-424-5p was measured using TaqMan MicroRNA Assays, with U6 as the internal control. The relative expression of the gene was calculated using 2^−ΔCT^ or 2^−ΔΔ CT^ method. The primer sequence was listed in Table 1.

**Table 1:** The primers used in this study.

Name	Sequences
HCG18 forward primer	5ʹ‑GGCGCGACACACAATACTC‑3ʹ
HCG18 reverse primer	5ʹ‑CCCTCCTCCTCCTTCTTCC‑3ʹ
miR-424-5p forward primer	5ʹ‑GGCAGCAGCAATTCATGT‑3ʹ
miR-424-5p reverse primer	5ʹ‑GAGAGGAGAGGAAGAGGGAA‑3ʹ
SOX9 forward primer	5ʹ‑CGGCCTCTACTCCACCTT‑3ʹ
SOX9 reverse primer	5ʹ‑AGACGGGTTGTTCCCAGT‑3ʹ
U6 forward primer	5ʹ-CTCGCTTCGGCAGCACA‐3ʹ
U6 reverse primer	5ʹ-GGATGGTGATGGTTTGGTAG‐3ʹ
GAPDH forward primer	5ʹ‐TCCTCTGACTTCAACAGCGACAC‐3ʹ
GAPDH reverse primer	5ʹ-CACCCTGTTGCTGTAGCCAAATTC‐3ʹ

### Western blot assay

Proteins were separated by 10% SDS-PAGE and transferred to PVDF membrane. TBST containing 5% skim milk was sealed at 37 °C for 2 h. The membranes were then incubated with primary antibodies including anti-Bcl-2 (#ab32124, 1/1000; Abcam), anti-Bax (#ab32503, 1/1000; Abcam), anti-Vimentin (#ab92547, 1/1000; Abcam), anti-E-cadherin (#ab231303, 1/1000; Abcam), anti-Cyclin D1 (#ab16663, 1/200; Abcam), anti-SOX9 (#ab185230, 1/1000; Abcam), anti-p-PI3k (#ab182651, 1/1000; Abcam), anti-PI3K (ab191606, 1/1000; Abcam), anti-p-AKT (#ab38449, 1/1000; Abcam), anti-AKT (ab131168, 1/1000; Abcam) at 4 °C overnight, the membrane was washed with TBST three times, five min each time. Then, the membranes were incubated with secondary antibodies for 2 h at room temperature, the membranes were washed with TBST three times, five min each time. ECL kit was used for chemiluminescence detection. Image Pro Plus 6.0 was used to analyze the relative expression of target protein, which was expressed by the ratio of density ratio to GAPDH.

### CCK-8 and EdU assays for cell viability and proliferation

For CCK-8 assay, transfected cells and the control group were seeded into 96-well plates with 4 × 10^3^ cells per well. 10 μL CCK-8 solution was added at 24, 48, 72 and 96 h, respectively. The plates were incubated for 4 h, and then the OD value of cells in each well was detected at 450 nm. EdU assay was performed to detect the cell proliferation. After transfection, cells were seeded into 24-well plates. When the cells grew to the logarithmic growth phase, 200 μL EdU staining solution (50 μmol/L) was added and incubated for 2 h, washed with PBS. Subsequently, fixed with 4% paraformaldehyde solution and incubated for 10 min. Added 200 μl of glycine (2 mg/mL) and incubate for 5 min, then wash with PBS on the shaker for 5 min. Added 100 μL PBS solution containing 0.5% TritonX-100 to each well, and incubated on shaker for 10 min, then wash with PBS twice, 5 min each time. Apollo was added for dark staining for 30 min, then incubation with DAPI staining solution at dark for 20 min. After PBS cleaning, photos were taken and counted under fluorescence microscope, and the results were analyzed. Total number of blue fluorescents labeled cells and proliferating red fluorescent labeled cells, cell proliferation rate (%) = number of red fluorescent cells/number of blue fluorescent cells × 100%.

### Colony formation assay

Cells after 48 h transfection were incubated to 6-well plates. The culture was stopped after cell colonies appeared. After fixed with formaldehyde and stained with crystal violet for 30 min, the clones with more than 50 cell colonies were counted under an inverted microscope.

### Cell apoptosis assay

Annexin V-FITC Apoptosis Assay Kit (Sigma-Aldrich, USA) was used to analyze apoptosis in QBC939 and HCCC9810 cells. After transfection, the cells were resuspended in 1 × Annexin V Binding Buffer (500 μL). Annexin V-FITC (5 μL) was added to fully mix. Cells were treated in darkness for 5 min. Cell apoptosis was analyzed by a flow cytometer (BD Biosciences, Franklin Lakes, NJ, USA). The apoptosis rate was calculated as the ratio of the number of apoptotic cells (Q2&Q3) to total cell number.

### Transwell assays

For migration: 200 μL cell suspension solution and 600 μL 1640 culture medium were added into the upper and lower chambers of Transwell chamber, respectively. Three wells were set in each group. After incubation for 72 hours, the cells were washed with PBS buffer, fixed with 95% alcohol, stained with 0.1% crystal violet, flushed and dried. The number of cell migration in 5 random fields in each group was counted under light microscope. The experiment was repeated three times. For invasion: a mixture of diluted glue (1640 medium and Matrigel glue were mixed in the ratio of 9:1) was added to the upper chamber surface of the bottom membrane of Transwell chamber after precooling at 4 °C. Cell suspension solution (200 μL) and 1640 medium (600 μL) were added into the upper and lower chambers of the chamber, respectively. Three wells were set in each group. The rest steps were the same as migration assay.

### Wound healing assay

The cells were seeded into 6-well plates and cultured in a 37 °C for 24 h. Cell transfection was carried out according to the above cell transfection method. When the cell density reached 80–90%, 200 μL tip was used for scratching operation. The cells were carefully washed by PBS for 2–3 times and cultured with 1640 containing 5% FBS. The scratch healing (closure width/original width) was observed dynamically by using inverted microscope at 0 h and 48 h respectively. The experiment was repeated for three times.

### Exosomes separation

Exosomes in cholangiocarcinoma cell culture supernatant were purified by differential ultracentrifugation. Briefly, cells were cultured in 1640 medium supplemented with 10% exosome-depleted FBS (SBI, CA, USA) for 48 h. Then cell culture supernatant was collected and spun at 300 g for 10 min under 4 °C to remove cells and 2000 g for 10 min under 4 °C to remove cellular debris. The resulting supernatant was followed by ultra-centrifugation at 10000 g for 30 min under 4 °C. Then the supernatant was ultra-centrifuged again at 100000 g for 70 min under 4 °C. The precipitate was the enriched exosomes and was resuspended in PBS. A small part of the precipitates were observed and photographed under transmission electron microscope (TEM), and the remaining precipitates were stored in Trizol LS (Invitrogen, USA) at −80 °C. Western blot was used to detect the specific protein biomarkers of exosomes.

### TEM observation and NTA (nanoparticle tracking analyzer)

A drop of exosomes suspension was loaded on the copper mesh of diameter 2 mm, and the liquid was gently absorbed from the edge of the copper mesh with filter paper. 2.5% glutaraldehyde was aimed to fix and alcohol solution was used to dehydration. After that, the sample was spilt into ultrathin slice and stained by uranyl acetate and lead citrate. Observed the morphology of exosomes under TEM and took photos under 80 kV. The NTA was applied via Zeta View system to observe the Brownian motion of the exosomes, and the size distribution was analyzed by Stokes-einstein equation.

### The cellular uptake of exosomes

Intracellular uptake of exosomes was stained with PKH26 (Sigma-Aldrich, USA) and the procedures were operated according to the instructions. The stained exosomes were added to the culture medium of HCCC9810 cells and incubated for 24 h, DAPI reagent (Invitrogen, USA) was applied to stain cell nuclear and finally a confocal microscope was used to take images.

### Fluorescence in situ hybridization (FISH) assay

QBC939 and HCCC9810 cells were selected to determine the co-localization of lncRNA HCG18 and miR-424-5p. CY3-labeled probe for lncRNA HCG18 (sequence:5ʹ-CY3-TCCCACCACACATCTTGCTGCTCCCTAAC-3ʹ) and DIG-labeled probe for miR-424-5p (sequence:5ʹ-DIG-TCAAAACATGAATTGCTGCTG-DIG-3ʹ) were used. The nuclear stained by DAPI were blue under ultraviolet excitation, and the positive expression was a kind of fluorescence labeled by corresponding luciferin. FAM (488) appears green, cy3 appears red. The detailed operations were followed under the official guideline.

### RNA immunoprecipitation

Magna RIP RNA-Binding Protein Immunoprecipitation Kit was used for RIP assay. To detected the miRNA binding to LncRNA, QBC939 cells were lysed in RIP lysis buffer and incubated with Biotin-coupled probe of HCG18 or olige probe which was pre-bound on magnetic beads. The purified miRNA was then subjected to qRT-PCR. To detected the LncRNA binding to miRNA, Biotin-coupled probe of miR-424-5p or Biotin-NC was processed through the same Protocol.

### Dual-Luciferase Reporter Assay

The wild-type (WT) and mutated sequences of HCG18 and 3ʹ UTR region of SOX9 were subcloned into the pGL3 Luciferase Reporter Vectors (Promega) to construct pGL3-HCG18-wt, pGL3-HCG18-mut, pGL3-SOX9-wt and pGL3-SOX9-mut. After the cells were grown to 70–80% fusion in the culture plate, cells were transfected with vectors containing wild-type or mutant HCG18 and Sox9 plasmids and miR-424-5p mimics or NC mimics using Lipofectamine 3000 at 37 °C for 48 h. The luciferase activity was detected by Dual-Luciferas Reporter Assay System and normalized to *Renilla* luciferase activity. The experiment was repeated for three times.

### Animal study

BALB/c nude mice were provided by the Vital River (Beijing, China). To establish subcutaneous tumor model, the cholangiocarcinoma cells (1 × 10^6^, 100 μL) stably expressing either HCG18-specific siRNA, HCG18 overexpression plasmids or control overexpression plasmids were subcutaneously injected into nude mice. Tumor volume was measured every seven days (V = 0.5 × length × width^2^). After 35 days, mice were sacrificed to weigh tumor tissues. For lung metastasis assessment, cells transfected with HCG18 knockdown and HCG18 overexpression plasmids were injected into BALB/c nude mice by tail vein, 6 weeks later, the pulmonary metastatic colonization of cholangiocarcinoma was monitored by non-invasive bioluminescence. Finally, mice were killed, tumor tissues were dissected. Metastatic nodules were counted and stained for hematoxylin-eosin to evaluate tumor metastasis.

### Hematoxylin-eosin (HE) staining

The lung metastasis was evaluated using HE staining. The mouse lung tissues were fixed with 4% paraformaldehyde, paraffin-embedded and cut into 5μm slices. Then the tissue sections were stained with hematoxylin and eosin. Finally, the slices were mounted and the histological changes were observed using a light microscope.

### Statistical analysis

SPSS 22.0 statistical software and GraphPad Prism 8 were used to analyze the experimental data. Measurement data were expressed as mean ± standard deviation ($\bar x$ ± s), *t*-test was used for comparison between two groups, and analysis of variance was used for comparison between multiple groups. *P* < 0.05 was seen as statistically significance.

## Results

### LncRNA HCG18 was highly enriched in cholangiocarcinoma tissues and cell lines

**Fig. 1:**
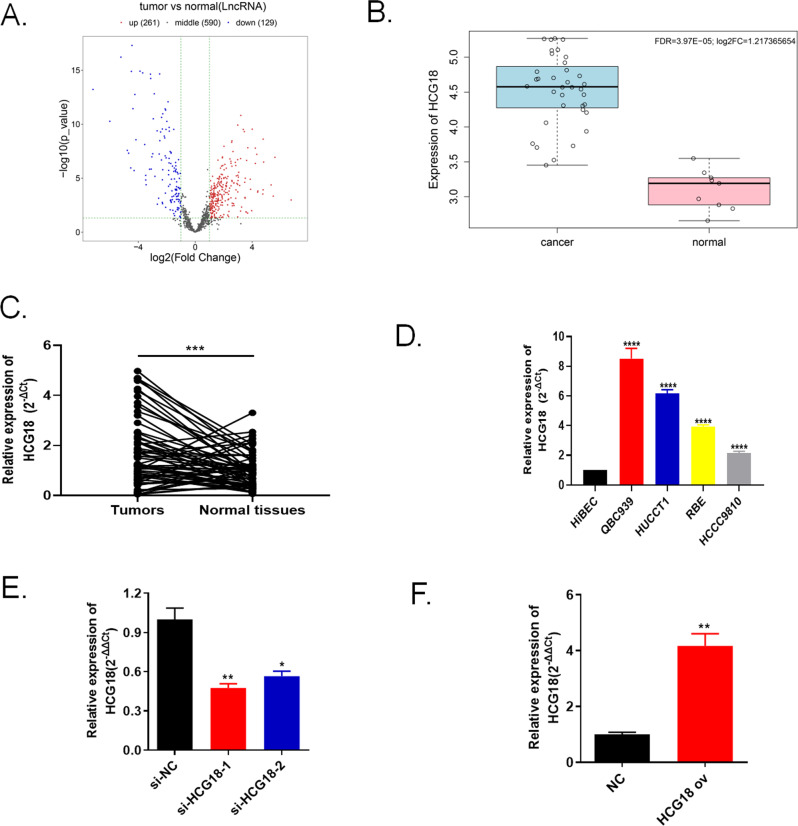
LncRNA HCG18 was highly expressed in cholangiocarcinoma tissues and cell lines. **A** Screening of differential expressed lncRNAs for cholangiocarcinoma through TCGA database. **B** Higher HCG18 level was found in cholangiocarcinoma tissues than in normal tissues based on TCGA database. **C** The expression of HCG18 in 60 pairs of cholangiocarcinoma tissues. ***P < 0.001 vs the normal tissue group. **D** The expression of HCG18 in four cancer cell lines (QBC939, HUCCT1, RBE, HCCC9810) and in normal human intrahepatic bile duct epithelial cell line HiBEC. ****P < 0.0001 vs the HiBEC group. **E** Interference plasmids (si-HCG18-1, si-HCG18-2) were transfected in QBC939 cells to down-regulate HCG18 detected by qRT-PCR, site 1 showed good effect, and subsequent functional experiments were performed on site 1. *P < 0.05, **P < 0.01 vs the si-NC group. **F** HCCC9810 cell line was transfected with overexpression plasmid (HCG18 ov) detected by qRT-PCR. **P < 0.01 vs the NC group. The data were presented as the mean ± standard deviation, the unpaired t test was used for comparisons between two groups, while one-way ANOVA followed by Tukey’s post hoc test was used for comparisons among multiple groups. All experiments were performed triplicate.

Through bioinformatics analysis in TCGA database to screen differential expressed lncRNAs, we found that higher HCG18 level in cholangiocarcinoma tumor tissue than in normal tissues (Fig. 1A, B). 60 pairs of tumor tissues and normal tissues of cholangiocarcinoma patients were collected and the data discovered that the expression of HCG18 was up-regulated in tumor samples (Fig. 1C). Additionally, qRT-PCR assay detected HCG18 expression level in four human cholangiocarcinoma cell lines (QBC939, HUCCT1, RBE, HCCC9810) and normal human intrahepatic bile duct epithelial cell line HiBEC. Figure 1D showed that HCG18 was enriched in cholangiocarcinoma cell lines compared with normal cell HiBEC. QBC939 cell line was transfected with interference plasmids (si-HCG18-1 and si-HCG18-2) to conduct HCG18 knockdown system, the HCG18 expression were lower level when they were given interference plasmids and si-HCG18-1 group (location 1) was chose for continue research due to the lower HCG18 level than si-HCG18-2 (Fig. 1E). In the same way, HCCC9810 was transfected with overexpression plasmid (HCG18 ov) to conduct HCG18 overexpression system and the HCG18 had higher expression in HCG18 ov group (Fig. 1F). These results indicated that HCG18 was highly enriched in cholangiocarcinoma tissues compared with normal samples.

### Knockdown of lncRNA HCG18 restrained cell growth, migration, invasion, EMT process and promoted cell apoptosis in cholangiocarcinoma cancer

**Fig. 2:**
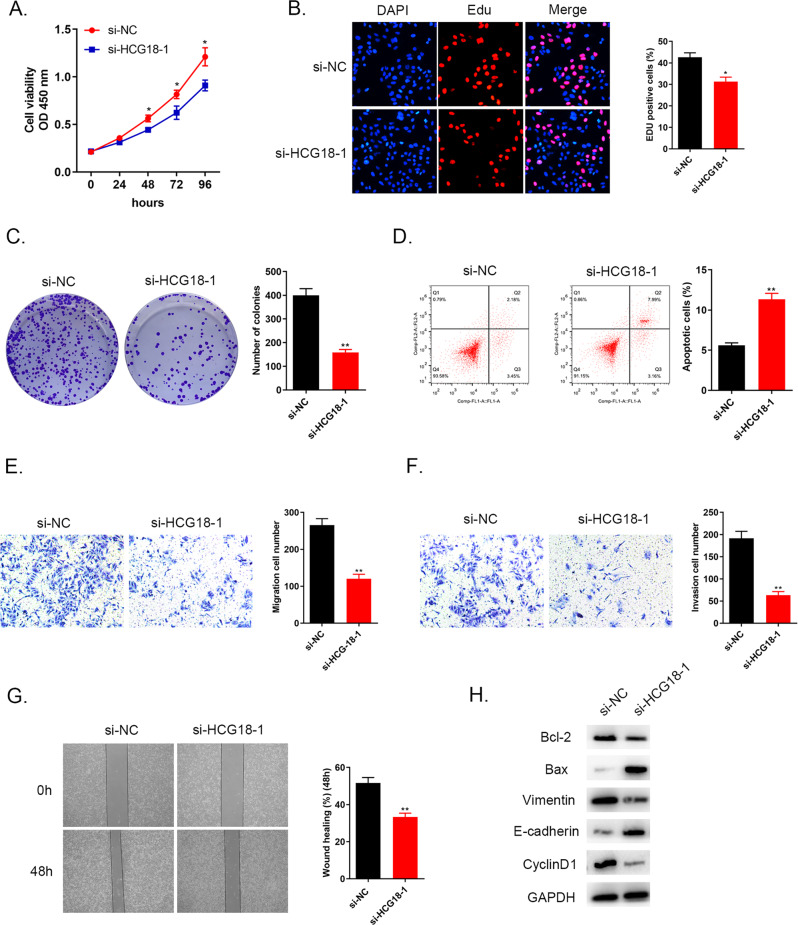
Down-regulation of HCG18 weakened cancer cells proliferation, migration and invasion, accelerated cell apoptosis. **A**–**C** In QBC939 cell line, cell viability and proliferation examined by CCK-8, EdU and colony formation assays was impaired after si-HCG18-1 transfection compared to si-NC group. **D** The reduced HCG18 level promoted cell apoptosis compared to si-NC group. **E**–**G** The results from transwell and wound healing assays demonstrated that the decreased HCG18 level could prevent migration and invasion. **H** Quantitation of apoptosis-related proteins (Bax and Bcl-2), EMT pathway biomarkers related to tumor transition (E-cadherin and vimentin) and cell cycle protein (CyclinD1) were determined by western blot. *P < 0.05, **P < 0.01 vs the si-NC group. The data were presented as the mean ± standard deviation, the unpaired *t* test was used for comparisons between two groups. All experiments were performed triplicate.

After conducting HCG18 knockdown group in QBC939 cells, several functional experiments were applied to evaluate HCG18 bioactivity in cholangiocarcinoma cancer cells. CCK-8, EdU and colony formation assays were aimed to detect cell proliferation (Fig. 2A–C). The results demonstrated that silencing HCG18 expression could inhibit cancer cells viability compared to the cells transfected with si-NC plasmid. Knockdown of HCG18 markedly promoted QBC939 cells apoptosis via flow cytometry assay (Fig. 2D). Wound healing and tranwell assays were performed to detect cells migration and invasion abilities. Figure 2E–G demonstrated that down-regulation of HCG18 level prevented cancer cells migration and invasion abilities in comparison of the si-NC group. Furthermore, western blot detected several apoptosis-related proteins (Bax and Bcl-2), EMT pathway biomarkers related to tumor transition (E-cadherin and Vimentin) and cell cycle protein (CyclinD1). The data in Fig. 2H revealed that knockdown HCG18 in si-HCG18-1 group could down-regulate apoptosis regulators Bcl-2 level and up-regulate Bax level, inhibit Vimentin expression and increase E-cadherin protein level and the decreasing CyclinD1 level was also observed in si-HCG18-1 group. Collectively, the evidence manifested that knockdown of lncRNA HCG18 could restrain cholangiocarcinoma cancer cells growth, migration, invasion and promote cancer cell apoptosis.

### Overexpression of lncRNA HCG18 contributed to cell proliferation, migration, invasion, EMT process and inhibited cell apoptosis in cholangiocarcinoma cancer

**Fig. 3:**
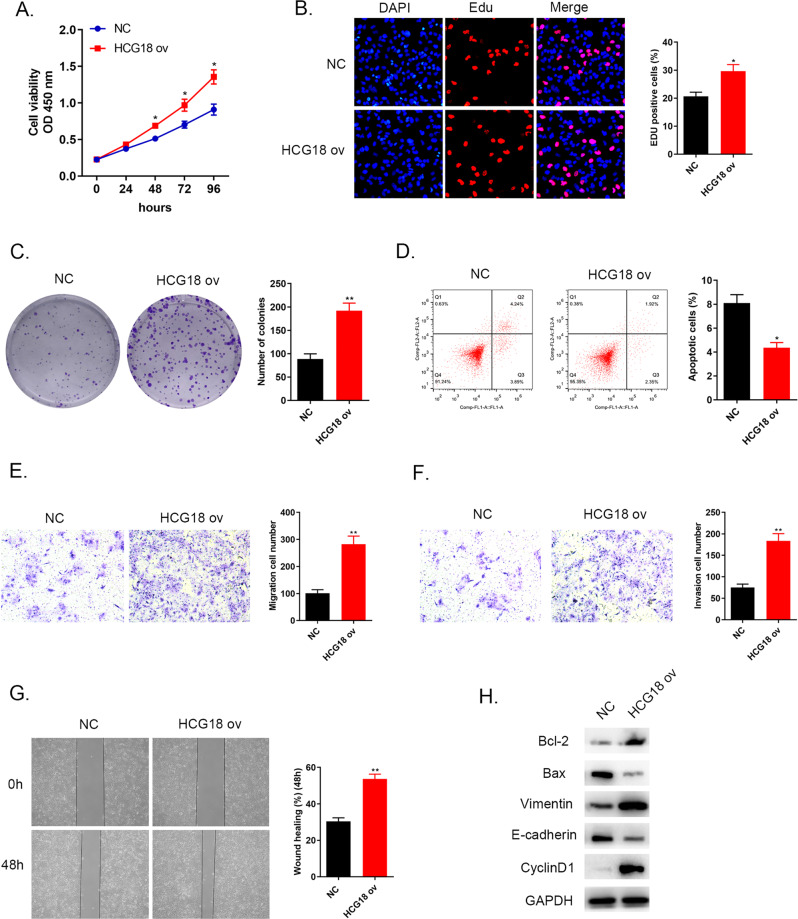
Overexpression of HCG18 contributed to cancer cells proliferation, migration and invasion, reduced cell apoptosis. **A**–**C** Cell proliferation examined by CCK-8, EdU and colony formation assays was strengthened after HCG18 overexpression in HCCC9810 cell line. **D** Through flow cytometry, cell apoptosis was prevented by high level of HCG18 compared to NC group. **E**–**G** Cell migration and invasion abilities evaluated by transwell and wound healing assays, indicated that the increased HCG18 level could fast cancer metastasis. **H** Quantitation of apoptosis-related proteins (Bax and Bcl-2), EMT pathway biomarkers related to tumor transition (E-cadherin and vimentin) and cell cycle protein (CyclinD1) were determined by western blot, revealed that high level of HCG18 could inhibit cell apoptosis, regulate EMT pathway. *P < 0.05, **P < 0.01 vs the NC group. The data were presented as the mean ± standard deviation, the unpaired *t* test was used for comparisons between two groups. All experiments were performed triplicate.

LncRNA HCG18 was overexpressed in HCCC9810 cell line using HCG18 overexpression plasmid. The following assays were determined to the influence of overexpression of HCG18 in cholangiocarcinoma. Figure 3A–C given the convincing evidence that higher HCG18 level accelerated cancer cell growth compared to NC group via CCK-8, EdU and colony formation assays. Flow cytometry results pointed that increasing HCG18 level reduced cancer cells apoptosis ability compared to control group (Fig. 3D). In the meanwhile, up-regulation of HCG18 also led to a positive impact on promoting cancer cells migration and invasion through wound healing and tranwell assays (Fig. 3E–G). For specific biomarkers associated with cell apoptosis, tumor metastasis and cell cycle detected by western blot, Fig. 3H showed that up-regulated apoptosis regulators Bcl-2 level and down-regulated Bax level, increasing Vimentin expression and decreasing E-cadherin protein level and the elevating CyclinD1 level were observed in HCG18 overexpression group compared to NC group. From all above results, overexpression of lncRNA HCG18 could contribute to cholangiocarcinoma cancer cells proliferation, migration and invasion.

### The effects of lncRNA HCG18 on tumor growth and lung metastasis of nude mice in vivo

**Fig. 4:**
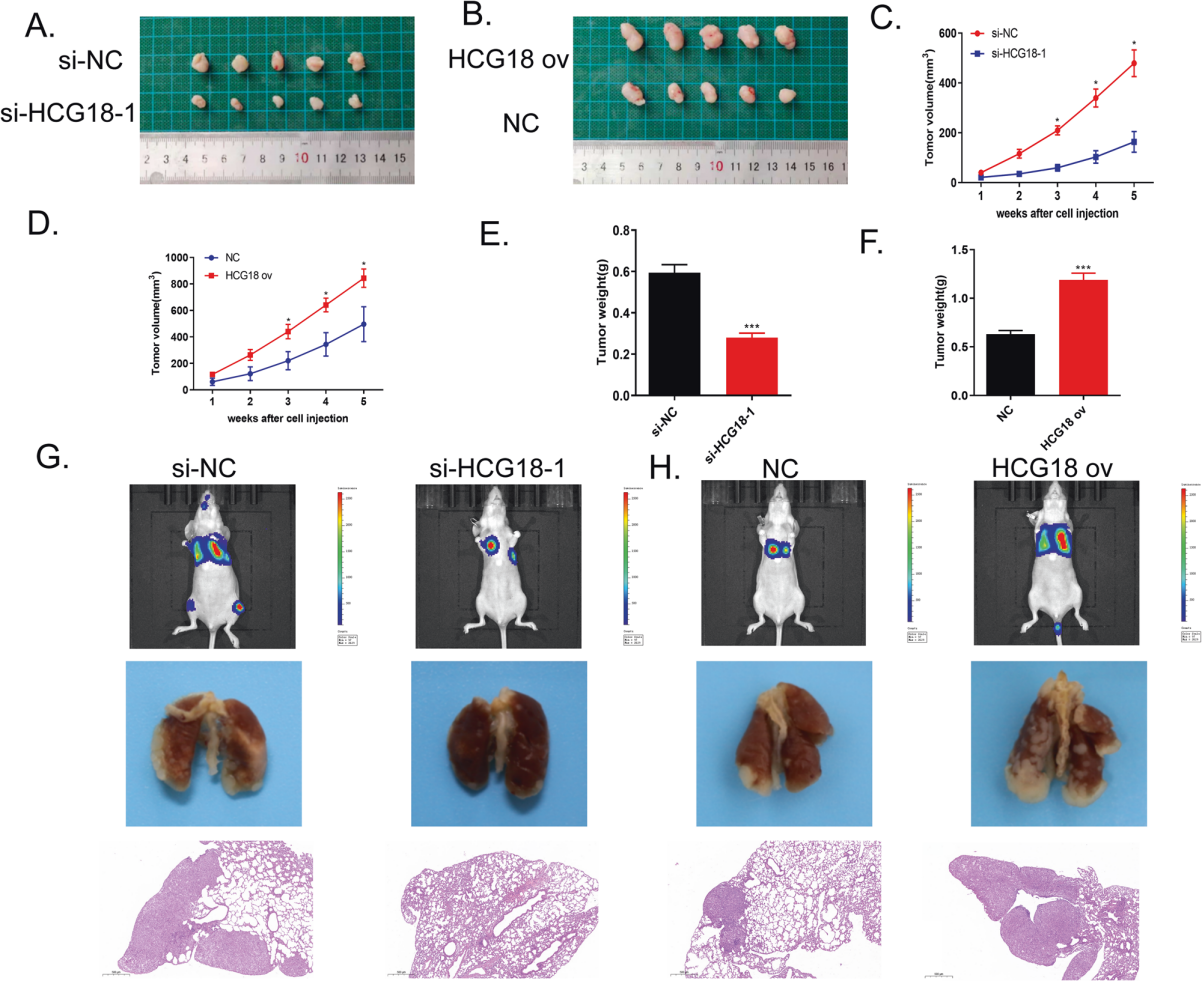
LncRNA HCG18 served as cancer-promoting gene, promoted tumor growth in vivo. **A** The general tumor image of the subcutaneous tumor in si-HCG18-1 and si-NC group. **B** The general tumor image of the subcutaneous tumor in HCG18 ov and NC group. **C** Tumor growth curve showed that knockdown HCG18 impeded tumor growth compared to si-NC group (The tumor volume was recorded every 7 day.) **D** The faster tumor growth rate was observed in HCG18 overexpression group compared to NC group (The tumor volume was recorded every 7 day.) **E** Knockdown HCG18 induced the lower average tumor weight in nude mice compared to si-NC group. **F** Higher HCG18 level resulted in the heavier average tumor weight compared to NC group after 35 days. **G** Fluorescence, general image and HE staining of lung metastases at 6 weeks, respectively. The results claimed that down-regulation of HCG18 inhibited tumor growth and metastasis in vivo. **H** Fluorescence, general image and HE staining of lung metastases at 6 weeks, respectively. The results claimed that up-regulation of HCG18 led to the faster tumor growth and metastasis in vivo. *P < 0.05, ***P < 0.001 vs the si-NC group. The data were presented as the mean ± standard deviation, the unpaired t test was used for comparisons between two groups.

The prior studies have addressed that lncRNA HCG18 positively promoted tumor growth, migration and invasion in vitro. Knockdown HCG18 effectively inhibited cancer cells proliferation, migration and invasion. Here, to give a more particular knowledge of HCG18 function, we established si-HCG18-1 or si-NC group stably transfected QBC939 cell line, and HCG18 ov or NC group transfected HCCC9810 cell line, which were all subcutaneously injected into nude mice. After 35 days injection, the image of xenograft tumors induced by si-HCG18-1 and si-NC group transfected QBC939 cells separated from mice was displayed in Fig. 4A. The si-HCG18-1 group showed the slower tumor volume growth rate than si-NC group (Fig. 4C). Knockdown HCG18 significantly reduced the average tumor weight compared to the si-NC group (Fig. 4E). The lung metastasis model was conducted through tail vein injection, demonstrating that, the prevention of HCG18 expression suppressed the lung metastasis of cholangiocarcinoma as compared with si-NC group under fluorescence and HE staining (Fig. 4G). On the contrary, the tumor volume and growth rate induced by HCG18 ov transfected cells were larger than NC group (Fig. 4B, D). The average tumor weight of HCG18 ov group stayed the same trend, indicating that overexpression of HCG18 promoted tumor growth (Fig. 4F). The lung metastasis results showed that increasing HCG18 level accelerated tumor metastasis in comparison of NC group (Fig. 4H). Taken together, lncRNA HCG18, served as a cancer-promoting gene, contributed to cholangiocarcinoma development and metastasis in vivo, which was consistent with the previous results in vitro.

### LncRNA HCG18 was transmitted through exosomes in cancer cells

**Fig. 5:**
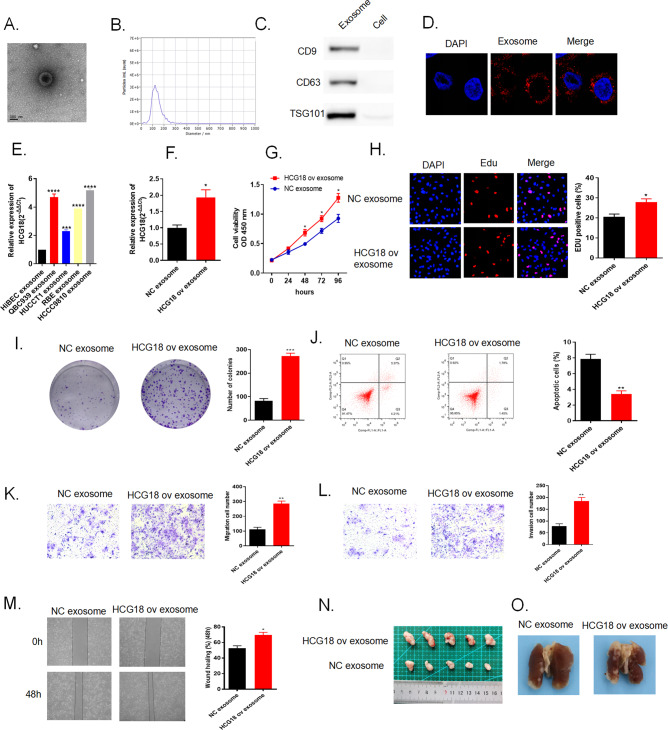
QBC939 cells secreted lncRNA HCG18 via exosomes. Overexpression of exosomal lncRNA HCG18 exerted its function in facilitating tumor development both in vitro and in vivo. HCG18 ov plasmid and NC were transfected in QBC939, and exosomes were extracted by differential hypercentrifugation. **A** The morphology of exosomes was observed by electron microscopy. **B** NTA was applied to observe the extracted particle size distribution. **C** Western blot detected by specific protein markers (CD9, CD63 and TSG101) of exosomes. **D** The extracted exosomes were labeled with PKH26 and then co-cultured with HCCC9810 cells. The phagocytosis effect was observed after 12 hours by confocal microscope. **E** The expression of HCG18 in cell exosomes. **F** After co-incubation, HCG18 expression detected by qRT-PCR in HCCC9810 cells transfected with HCG18 ov exosome was higher than that in NC group. **G**–**I** Cell proliferation rate was promoted by HCG18 ov exosome compared to NC exosome group through CCK-8, EdU and colony formation assays. **J** Flow cytometry assay showed that cell apoptosis was restrained by HCG18 ov exosome compared to NC exosome group. **K**–**M** Transwell and wound healing assays results indicated that cancer cell migration and invasion abilities were intensified by HCG18 ov exosome compared to NC exosome group. **N**, **O** General tumor tissues images meant exosomal HCG18 overexpression could enhance tumor growth and metastasis rate in vivo. *P < 0.05, **P < 0.01, ***P < 0.001, ****P  < 0.0001 vs the NC exosome group. The data were presented as the mean ± standard deviation, the unpaired *t* test was used for comparisons between two groups.

As mentioned before, lncRNAs can be contained in exosomes to release to the surrounding cells therefore mediating different biological activity. Herein, HCG18 ov and NC plasmids were transfected in QBC939 cell line, served as our study subject. Extracellular exosomes of tumor cells were isolated and purified by differential centrifugation following the standard methods. The morphological character and size distribution of the separated exosomes were observed through Transmission Electron Microscope (TEM) and Nanosight particle tracking analysis (Fig. 5A, B). From the results, we identified the vesicles diameter was about 100–150 nm, which were consistent with literature data. Subsequently, we applied western blot to detect the specific markers [26, 27] (CD63, CD9, and TSG101) to identify exosomes (Fig. 5C). Through these findings, we confirmed the isolated particles are exosomes. To vertify the delivery of exosomal lncRNA, the exosomes were labeled with membrane phospholipid dye PKH26 and co-incubated with HCCC9810 cells. After 12 h, the recipient HCCC9810 cells displayed the distinct uptake of labeled exosomes around the nucleus by laser confocal microscope (Fig. 5D). Thus, cholangiocarcinoma cells transmit HCG18 to the surrounding cancer cells through exosomes. We also detected the expression of HCG18 in exosomes. The result indicated that HCG18 was highly expressed in cholangiocarcinoma cell exosomes (Fig. 5E). In the meantime, qRT-PCR showed that HCG18 level was much higher in HCCC9810 cells transfected with HCG18 ov exosomes than NC exosomes (Fig. 5F). Collectively, the data demonstrated that LncRNA HCG18 was transmitted through exosomes in cancer cells.

### Overexpression of exosomal lncRNA HCG18 exerted its function in facilitating tumor development both in vitro and in vivo

We have already established co-culture model of HCG18 ov exosomes and HCCC9810 cells to observe the entrance of exosomal lncRNA HCG18. Next, we investigated the impact of exosomal lncRNA HCG18 on cancer cells development via several functional experiments. CCK-8 assay (Fig. 5G), EdU assay (Fig. 5H) and colony formation assay (Fig. 5I) collectively illustrated cancer cell viability and proliferation. Results claimed that compared to NC exosome group, the viability and proliferation of cells transfected with HCG18 ov exosomes was promoted. Cell apoptosis ability was measured by flow cytometry and Fig. 5J pointed that transfected with HCG18 ov exosomes could restrain cancer cell apoptosis compared to NC group. Transwell assays and wound healing assay jointly explained that enhanced migration and invasion abilities were observed in HCCC9810 cells co-cultured with HCG18 ov exosomes compared to NC group (Fig. 5K–M). In vivo animal study showed that tumor growth and metastasis was promoted in HCG18 exosome group compared to NC group (Fig. 5N, O). All results suggested that overexpression of exosomal lncRNA HCG18 exerted its function in facilitating tumor development both in vitro and in vivo.

### LncRNA HCG18 served as a sponge of miR-424-5p in cholangiocarcinoma cells

**Fig. 6:**
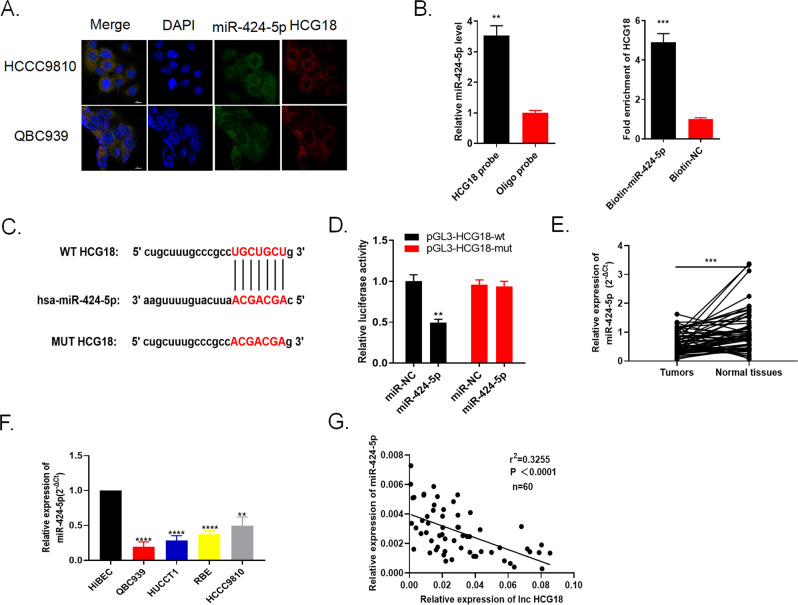
lncRNA HCG18 targeted to miR-424-5p with negative modulation. **A** FISH assay indicated that HCG18 and miR-424-5p co-localized in cytoplasm. **B** RIP experiment showed that HCG18 directly targeted miR-424-5p. **C** Starbase database indicated there was a complementary sequence between HCG18 and miR-424-5p. **D** The luciferase report experiment supported the direct relationship between HCG18 and miR-424-5p. **E** The level of miR-424-5p in 60 pairs of cholangiocarcinoma tissues was detected by qRT-PCR. **F** The level of miR-424-5p in 4 cancer cell lines (QBC939, HUCCT1, RBE, HCCC9810) and in normal human intrahepatic bile duct epithelial cell line HiBEC was detected by qRT-PCR. **G** MiR-424-5p was negatively related to HCG18. **P < 0.01, ***P < 0.001, ****P < 0.0001 vs the control group. The data were presented as the mean ± standard deviation, the unpaired *t* test was used for comparisons between two groups. All experiments were performed triplicate.

In order to further understand the function and regulatory role of lncRNA HCG18, it is necessary to understand its localization and distribution in cancer cells. FISH results in Fig. 6A stated that HCG18 and miR-424-5p was co-localized in the cytoplasm of HCCC9810 and QBC939 cells. RIP assay showed the abundant enrichment of HCG18 and miR-424-5p in the precipitates of anti-Ago2, which indicated that HCG18 bound with miR-424-5p in cholangiocarcinoma cells (Fig. 6B). Through bioinformatics analysis, we found the possible complementary sequences between HCG18 and miR-424-5p in Starbase (Fig. 6C). Dual luciferase reporter assay proved that there was the direct interaction between HCG18 and miR-424-5p. The luciferase activity of pGL3-HCG18-wt cells was reduced treated with miR-424-5p mimics when compared to miR-NC group while there was no significant change in luciferase activity of pGL3-HCG18-mut group (Fig. 6D). After that, we detect the expression of miR-424-5p in 60 pairs of cholangiocarcinoma and normal tissues. Figure 6E manifested that reductive miR-424-5p level was detected in tumor tissues compared to normal tissues. qRT-PCR assay detected miR-424-5p level expression level in 4 cholangiocarcinoma cell lines (QBC939, HUCCT1, RBE, HCCC9810) and normal human intrahepatic bile duct epithelial cell line HiBEC. Figure 6F showed that miR-424-5p was repressed in cholangiocarcinoma cell lines compared with normal cell HiBEC. Pearson’s correlation analysis revealed there was negative correlation between HCG18 and miR-424-5p expression (Fig. 6G). All the results declared that miR-424-5p was direct target of HCG18 with negative regulation and miR-424-5p was down-regulated both in cholangiocarcinoma tissues and cell lines.

### The inhibition of miR-424-5p reversed the suppression of cancer cells induced by lncRNA HCG18 knockdown

**Fig. 7:**
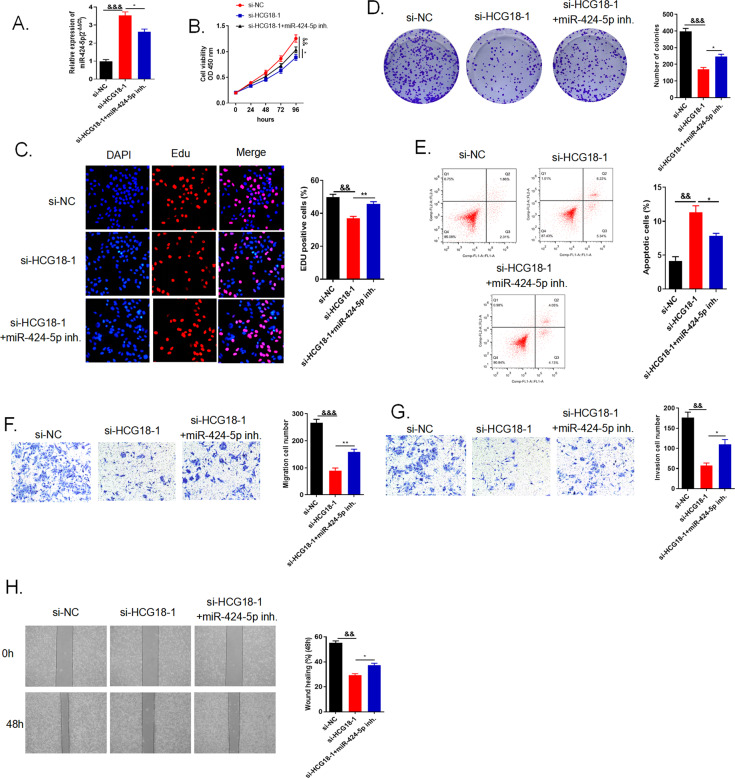
The inhibition of miR-424-5p reversed the suppression of cancer cells induced by lncRNA HCG18 knockdown. **A** The relative expressions of miR-424-5p were evaluated via qRT-PCR in QBC939 cells transfected with the si-NC, si-HCG18-1 or si-HCG18-1 + miR-424-5p inhibitor, respectively. **B**–**D** CCK-8, EdU and colony formation assays were applied to determine cell proliferation capacity. After adding miR-424-5p inhibitor, cell viability and proliferation was enhanced compared to si-HCG18-1 group. **E** Flow cytometry said miR-424-5p inhibitor could suppress cell apoptosis. **F**–**H** Cell metastasis was evaluated by transwell and wound healing assays and the data displayed that si-HCG18-1 + miR-424-5p inhibitor could accelerate cell migration and invasion compared to si-HCG18-1 group. && < 0.01, &&& < 0.001 vs the si-NC group; *P < 0.05, **P < 0.01 vs the si-HCG18-1 group. The data were presented as the mean ± standard deviation, the unpaired t test was used for comparisons between two groups. All experiments were performed triplicate.

To explore miR-424-5p biological function in cancer development and biological interaction with HCG18, we conducted several rescue experiments in three groups with different transfections in QBC939 cells. QRT-PCR was measured for miR-424-5p, miR-424-5p was declined in si-HCG18 + miR-424-5p inhibitor group compared to si-HCG18 group (Fig. 7A). After the co-transfection of miR-424-5p inhibitor and si-HCG18 in QBC939 cells, we observed that cell proliferation capacity was promoted through CCK-8, EdU and colony formation assays compared to si-HCG18 group (Fig. 7B–D). Moreover, adding miR-424-5p inhibitor in si-HCG18 group could restrain cancer cell apoptosis compared to si-HCG18 group (Fig. 7E). In the same way, knockdown HCG18 could mitigate cancer cell migration and invasion while add miR-424-5p inhibitor could alter this result, promoting cancer cell migration and invasion (Fig. 7F–H). Thus, we concluded that miR-424-5p played the suppressive role in cholangiocarcinoma development and the inhibition of miR-424-5p reversed the suppression of cancer cells induced by lncRNA HCG18 knockdown.

### MiR-424-5p negatively targeted SOX9

**Fig. 8:**
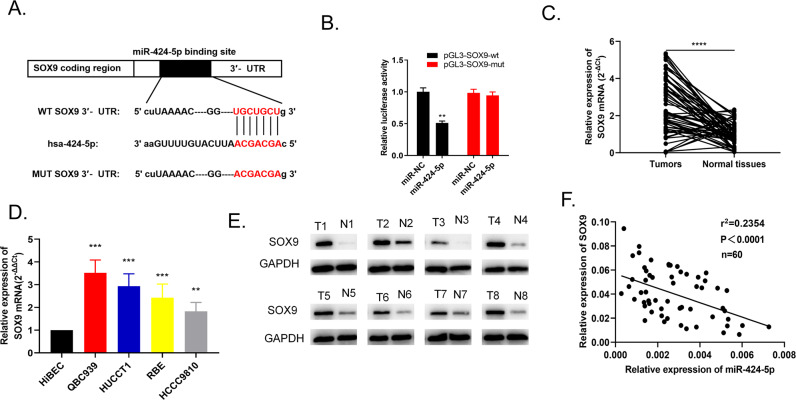
MiR-424-5p could bind with SOX9 with negative regulation. **A** The database indicates that there was complementary sequence between SOX9 and miR-424-5p. **B** Dual luciferase report assay exhibited this direct interaction. **C** SOX9 mRNA level in 60 pairs of tumor tissues were detected by qRT-PCR. **D** SOX9 mRNA level in four cancer cell lines (QBC939, HUCCT1, RBE, HCCC9810) and in normal human intrahepatic bile duct epithelial cell line HiBEC was detected by qRT-PCR. **E** SOX9 protein level in 8 pairs of tissues was detected by western blot. **F** SOX9 and miR-424-5p expression were negatively correlated. **P < 0.01, ***P < 0.001, ****P < 0.0001 vs the control group. The data were presented as the mean ± standard deviation, the unpaired t test was used for comparisons between two groups. All experiments were performed triplicate.

According to bioinformatic prediction, Fig. 8A displayed that there was a putative binding site between miR-424-5p and SOX9. Dual luciferase reporter assay further confirmed this direct interaction. The results showed that the miR-424-5p mimics treated group obviously restrained the luciferase activity of pGL3-SOX9-wt cells when compared to miR-NC treated. However, the luciferase activity of pGL3-SOX9-mut cells remained no statistical change (Fig. 8B). QRT-PCR determined SOX9 mRNA level in 60 pairs of cholangiocarcinoma tissues and cell lines (QBC939, HUCCT1, RBE, HCCC9810, HiBEC). The data exhibited that SOX9 mRNA level was excessive both in tumor tissues and cancer cells when compared to the normal group (Fig. 8C, D). In addition, the SOX9 protein expression level was assessed via western blot in eight pairs of tumor tissues. Figure 8E manifested that the level of SOX9 was overexpressed in comparison of normal tissues and Pearson’s correlation analysis revealed there was negative correlation between miR-424-5p and SOX9 expression (*r*^2^ = 0.2354, *P* < 0.0001) (Fig. 8F). From above results, we concluded that miR-424-5p could directly bind with SOX9 and negatively regulated SOX9 expression.

### Overexpression of SOX9 altered the repression of cancer cell mediated by lncRNA HCG18 knockdown through PI3K/AKT pathway

**Fig. 9:**
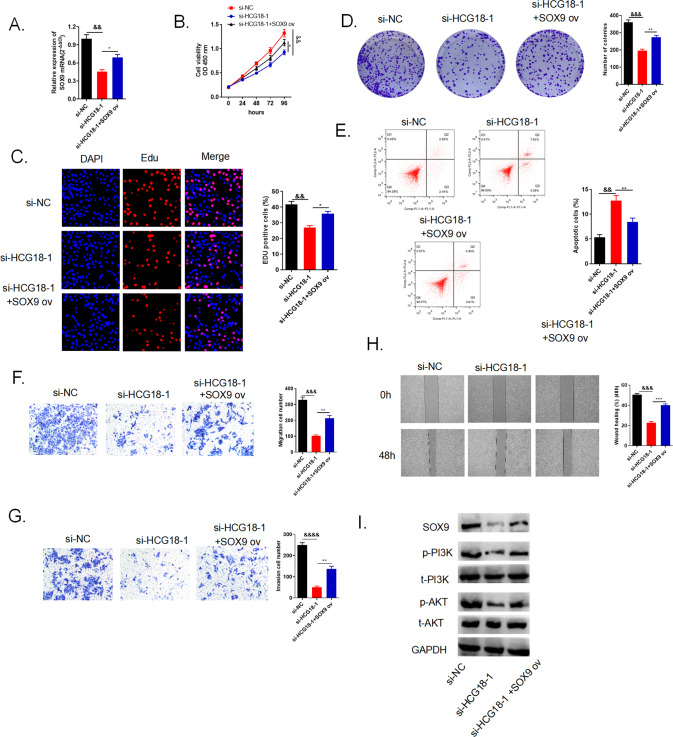
Overexpression of SOX9 altered the inhibition of cancer cell promotion mediated by lncRNA HCG18 knockdown through PI3K/AKT pathway. **A** The relative expressions of SOX9 mRNA were evaluated via qRT-PCR in QBC939 cells transfected with the si-NC, si-HCG18-1 or si-HCG18-1 + SOX9 ov, respectively. **B**–**D** CCK-8, EdU and colony formation assays were applied to determine cell proliferation capacity. Cell viability and proliferation was enhanced in si-HCG18-1 + SOX9 ov compared to si-HCG18-1 group. **E** Flow cytometry said higher SOX9 mRNA level could restrain cell apoptosis. **F**–**H** Cell metastasis was evaluated by transwell and wound healing assays, the evidence showed that SOX9 overexpression could speed up cell migration and invasion compared to si-HCG18-1 group. (I) Western blot examined the related protein levels in PI3K/AKT pathway. && < 0.01, &&& < 0.001, &&&& < 0.0001 vs the si-NC group; *P < 0.05, **P < 0.01, ***P < 0.001 vs the si-HCG18-1 group. The data were presented as the mean ± standard deviation, the unpaired t test was used for comparisons between two groups. All experiments were performed triplicate.

We established three different transfections groups in QBC939 cell line to explore the biological role of SOX9 in cancer cell growth. QRT-PCR was applied to measure the transfection efficiency. The increased SOX9 mRNA level was in the group with si-HCG18-1 and SOX9 ov transfections simultaneously when compared to si-HCG18-1 group (Fig. 9A). Through CCK-8, EdU and colony formation assays, we found that increasing SOX9 level in si-HCG18-1 and SOX9 ov co-transfection group could facilitate cancer cell proliferation when compared to si-HCG18-1 group (Fig. 9B–D). Cell apoptosis was also impeded by SOX9 overexpression when compared to si-HCG18-1 group (Fig. 9E). What’s more, transwell and wound healing assays revealed that up-regulated SOX9 level could reverse the restrain of cancer cells migration and invasion brought by silencing HCG18 (Fig. 9F–H). Classical PI3K/Akt signaling pathway is proved to relate with proliferation, differentiation and apoptosis, and numerous researches have reported PI3K/Akt related to cancer development [28]. We applied western blot to detect the specific proteins related to PI3K/AKT pathway. Figure 9I displayed that when compare to si-HCG18-1 group, phosphorylation of PI3K (p-PI3K) and AKT (p-AKT) was enhanced in si-HCG18-1 and SOX9 ov co-transfection group while total PI3K (t-PI3K) and AKT (t-AKT) were unchanged. In a word, the results demonstrated that overexpression of SOX9 altered the repression of cancer cell mediated by lncRNA HCG18 knockdown through PI3K/AKT pathway.

## Discussion

Cholangiocarcinoma is a highly aggressive tumor disease with malignant death rate. It’s urgent to identify specific biomarkers for cholangiocarcinoma treatment and diagnosis. Previous studies that have noted the importance of lncRNAs in cancer and the following mechanism was fully explored. Overexpression of lncRNA ZFAS1 was reported to be a diagnosis feature in ESCC tissues [29]. Elevated lncRNA AGAP2-AS1 level was observed in prostate cancer [30]. The role of lncRNA HCG18 was previously reported to be a cancer-promoting gene, related to bladder cancer, papillary thyroid cancer, etc. [31, 32].

Herein, a key primary conclusion of this study was that lncRNA HCG18 was up-regulated in cholangiocarcinoma tumor tissues and cancer cell lines compared to normal groups. Through several functional experiments CCK-8, colony formation, EdU, transwell, flow cytometry, wound healing assays and animal study, we identified the biological function of HCG18 in cholangiocarcinoma. Increased HCG18 level could contribute to cancer proliferation, migration and invasion both in vivo and in vitro, reduce cancer cell apoptosis. In addition, we found cancer cell-secreted exosomes transitted HCG18 and accelerated surrounding tumor cells growth and metastasis. It has been established that exosomes containing various lncRNAs carried out functional activity and delivered information associated with tumor development. For instance, exosomal lncRNA 91H were found to promoted colorectal cancer viability and transmission [33]. ESCC cells exosomes transferred oncogene lncRNA ZFAS1 to the surrounding cells to facilitate tumor growth [34]. These results suggested that HCG18 was a cancer-promoting gene for cholangiocarcinoma.

In mounting researches, lncRNAs usually bind with miRNAs as sponge to regulate cancer development in ceRNA network. lncRNA GAS5 was negatively modulated by miR-21 in breast cancer [35]. LINC00473 influence prostate cancer growth by sponging miR-195-5p/SEPT2 [36]. In this study, we confirmed HCG18 directly interacted with miR-424-5p through FISH, RIP and luciferase reporter assays and HCG18 inhibited miR-424-5p expression. The inhibition of miR-424-5p reversed the suppression of cholangiocarcinoma cancer cells proliferation, migration and invasion induced by HCG18 knockdown. In addition, the data demonstrated that HCG18 might sponge miR-424-5p to enhance the tumorigenesis, metastasis in vivo and miR-424-5p was the possible tumor suppressor. SOX9, Sex determining region Y-box 9, was reported to be a potential tumor diver, regulated by various miRNAs and reactions like methylation, phosphorylation, and acetylation [25]. Through targeting SOX9, miR-145 negatively modulated chondrogenic differentiation [37]. PI3K/AKT pathway was closely related to tumor proliferation, differentiation and apoptosis. Numerous studies found that activation of PI3K/AKT could contribute to multiple tumorigenesis, like glioma, breast cancer [38, 39]. Here, we discovered that miR-424-5p could target SOX9 with negative correlation. Furthermore, through corresponding functional assays, indicated that SOX9 was an oncogene, overexpression of SOX9 altered the inhibition of cancer cell promotion mediated by lncRNA HCG18 knockdown through PI3K/AKT pathway.

Although we have fully demonstrated that HCG18 was potential marker for cholangiocarcinoma and contributed to tumor growth and metastasis through mediating miR-424-5p/SOX9 axis through PI3K/AKT pathway. There are still limitations for further study. MiR-424-5p may not be the only target of HCG18 and the associated regulation loop may involve more complicated. The following researches need to continue.

## Conclusion

In conclusion, in this study, we firstly found a novel marker lncRNA HCG18 overexpression in cholangiocarcinoma. Elevated HCG18 level could contribute to cancer proliferation, migration and invasion both in vivo and in vitro, reduce cancer cell apoptosis. In addition, we found cancer cell-secreted exosomes transitted HCG18 and accelerated surrounding tumor cells growth and metastasis. MiR-424-5p, served as tumor suppressor, was a direct target of HCG18 and was negatively regulated by HCG18. More specificly, miR-424-5p target to SOX9 contributed to cholangiocarcinoma growth and metastasis through mediating miR-424-5p/SOX9 axis through PI3K/AKT pathway.

## Supplementary information

WB Original

qPCR Original

## Data Availability

All data generated and/or analyzed during this study are included in this published article.
