# Supplementary material for: Exosomal lncRNA HCG18 contributes to cholangiocarcinoma growth and metastasis through mediating miR-424-5p/SOX9 axis through PI3K/AKT pathway
Source: Cancer Gene Ther. 2023 Feb 28;30(4):582–95. doi: 10.1038/s41417-022-00500-2 (PMC10104778; doi:10.1038/s41417-022-00500-2)
Supplement: Supplementary file 1 — WB Original [file 41417_2022_500_MOESM1_ESM.pdf]

figure 2H and 3H

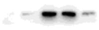

Bax

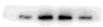

E-cadherin

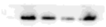

Bcl-2

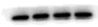

GAPDH

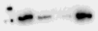

Cyclin d1

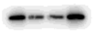

Vimentin

figure 8E

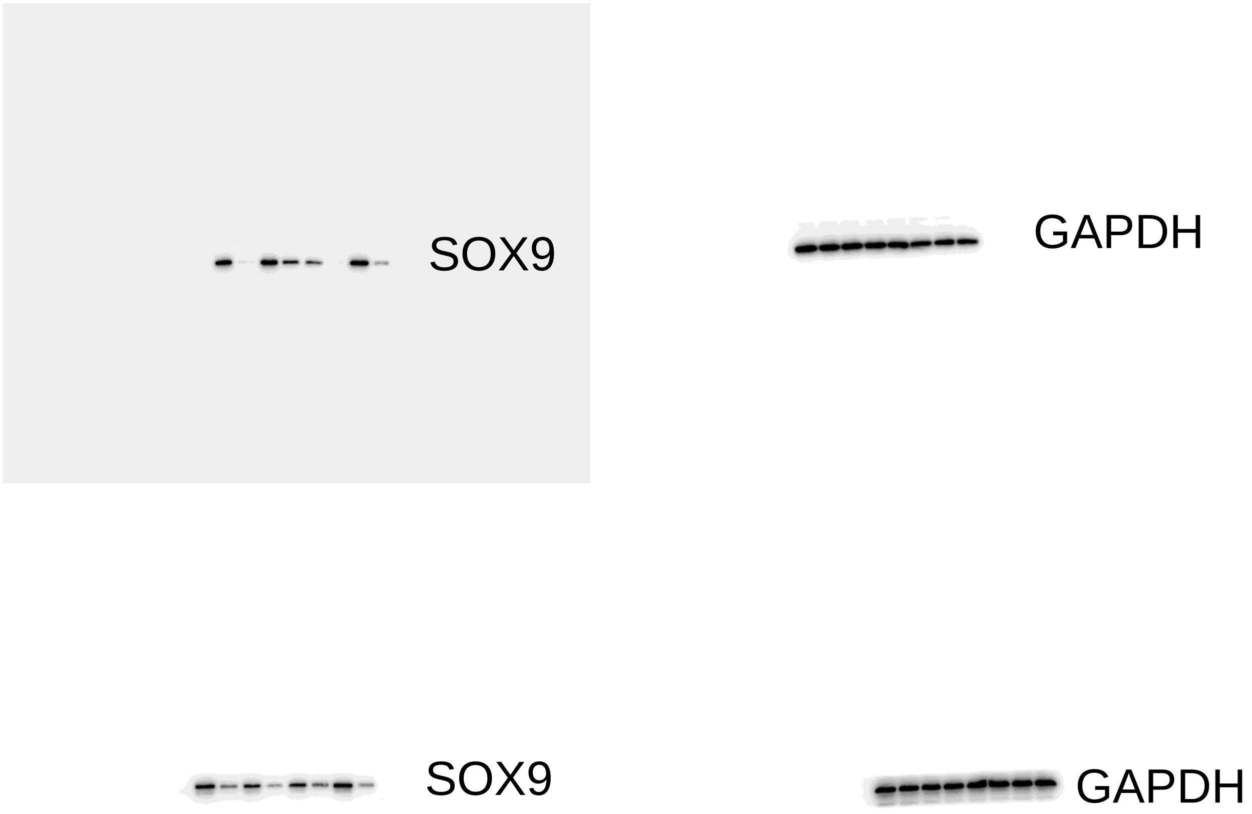

figure 9I

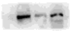

SOX9

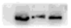

p-PI3K

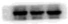

t-PI3K

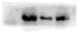

p-AKT

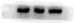

t-AKT

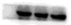

GAPDH
